# Medication error affecting newborns' sight: a national response

**Published:** 2019-09-10

**Authors:** Nyawira Mwangi, Michael Mbee Gichangi

**Affiliations:** 1 Research Fellow: London School of Hygiene and Tropical Medicine, London, UK.; 2 Head: Ophthalmic Services Unit: Ministry of Health, Nairobi, Kenya.

**There have been several reports in Kenya of the wrong use of chlorhexidine digluconate solution 7.1% in the eyes of babies, resulting in chemical injury. Nyawira Mwangi spoke with Michael Gichangi, lead ophthalmologist at Kenya's Ministry of Health, about this problem.**

**Figure:**
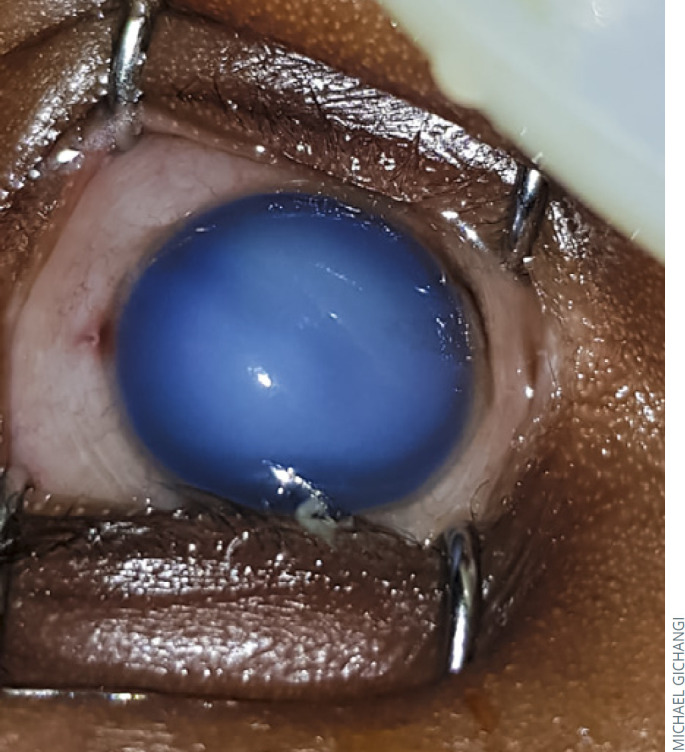
Chlorhexidine 7.1% is toxic to the cornea. KENYA

Chlorhexidine is an antiseptic recommended by the World Health Organisation for use on the umbilical cord in order to prevent neonatal sepsis. It must be applied when the cord is cut and for six days afterwards. The packaging used resembles eye drop bottles, and reports indicate that some mothers therefore confuse the antiseptic with eye drops and instil it in their babies' eyes. At the concentration of 7.1%, chlorhexidine is toxic to the cornea and can cause permanent visual loss. This has been reported in other countries previously.^1^

## Q: What is the situation now?

Publicity and discussion of this issue in mainstream and social media has allowed the public, the eye care community, pharmacists, paediatricians, eye care managers and other stakeholders to become aware of this important issue.

Patient safety is receiving growing attention in Kenya, and eye care providers are on the alert. We are discussing the issue at conferences, and we are also examining the broad factors that have contributed to the error, including manufacturing, packaging, importing and dispensing, as well as the way errors are reported and handled. This has strengthened the health system, as the processes of reporting medication errors and investigating them has now become clearer.

## Q: How are you dealing with this problem?

We are working with Kenya's national Pharmacy and Poisons Board (PPB), which is receiving the reports and investigating the errors, and we are also having conversations with the manufacturers about changing the packaging to avoid confusion with eye drops. There is also a need to educate the mother or carer when dispensing the drugs; it is not enough to tell them: “These are your drugs to take home.” It is important to explain how to use the medication. We are encouraging reporting as well as quick diagnosis, treatment and counselling.

We are therefore training health care workers to do the following:

- When dispensing chlorhexidine, inform mothers that the antiseptic is for the umbilical cord only, not the eye.
- Advise mothers to seek immediate help if such an incident occurs. If near a health facility, they should rush the baby for prompt skilled health care. If far from a health facility, they should irrigate the eye with clean running tap water.
- Provide emergency care to any babies affected: irrigate the eye with normal saline, apply a topical anaesthetic, give an oral painkiller and refer the baby to an ophthalmologist as soon as possible.
- Report all incidents. The PPB has requested that health workers report each incident of this error on a specified reporting form in order to generate evidence for action.

## Q: What are the challenges with medical errors in Kenya, more broadly?

Many medical errors are not reported. Alternative approaches are needed to identify a much higher proportion of the errors that actually occur and go unreported.

There are many barriers that currently prevent the reporting of errors. To increase our opportunities to learn from errors, the following three changes are needed:

1. A more open culture of discussing errors, including discussing them at professional forums.
2. Increasing awareness amongst health workers about the reporting mechanism of the Pharmacy and Poisons Board.
3. Adequate investigation of the root causes of errors.

**Figure:**
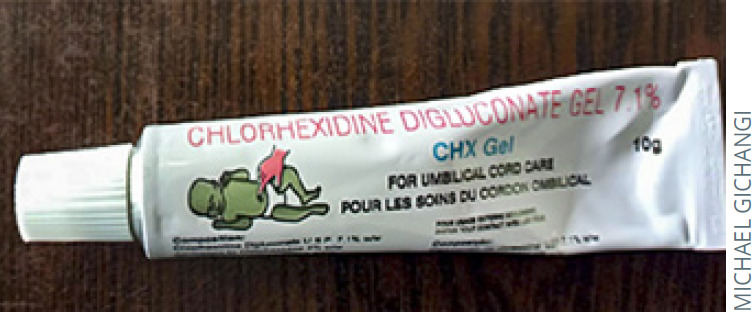
Chlorhexidine ointment can be mistaken for eye ointment.

## Q: Can technology make a difference in reporting or dealing with medical errors?

mHealth applications might increase efficiency of reporting. In this instance, we have used mHealth and social media to publicise and discuss the issue within the eye care community.
